# Depletion of Cutaneous Macrophages and Dendritic Cells Promotes Growth of Basal Cell Carcinoma in Mice

**DOI:** 10.1371/journal.pone.0093555

**Published:** 2014-04-01

**Authors:** Simone König, Frauke Nitzki, Anja Uhmann, Kai Dittmann, Jennifer Theiss-Suennemann, Markus Herrmann, Holger M. Reichardt, Reto Schwendener, Tobias Pukrop, Walter Schulz-Schaeffer, Heidi Hahn

**Affiliations:** 1 Institute of Human Genetics, University Medical Center, Goettingen, Germany; 2 Institute of Cellular and Molecular Immunology, University Medical Center, Goettingen, Germany; 3 Department of Radiation Oncology, University Medical Center, Goettingen, Germany; 4 Institute of Molecular Cancer Research, University of Zurich, Zurich, Switzerland; 5 Department of Hematology and Oncology, University Medical Center, Goettingen, Germany; 6 Department of Neuropathology, University Medical Center, Goettingen, Germany; Ohio State University Medical Center, United States of America

## Abstract

Basal cell carcinoma (BCC) belongs to the group of non-melanoma skin tumors and is the most common tumor in the western world. BCC arises due to mutations in the tumor suppressor gene *Patched1* (*Ptch*). Analysis of the conditional *Ptch* knockout mouse model for BCC reveals that macrophages and dendritic cells (DC) of the skin play an important role in BCC growth restraining processes. This is based on the observation that a clodronate-liposome mediated depletion of these cells in the tumor-bearing skin results in significant BCC enlargement. The depletion of these cells does not modulate Ki67 or *K10* expression, but is accompanied by a decrease in collagen-producing cells in the tumor stroma. Together, the data suggest that cutaneous macrophages and DC in the tumor microenvironment exert an antitumor effect on BCC.

## Introduction

BCC is the most commonly diagnosed tumor among people in the western world and arises due to mutations in the tumor suppressor gene *Ptch*. Although BCC rarely metastasizes it can cause significant morbidity due to local aggressiveness and recurrences [1]. Interestingly, approximately 20% of BCCs show signs of spontaneous regression [2]. BCC regression is associated with significantly increased numbers of T cells and the expression of the IL-2 receptor, which is an early activation marker for T cells [3]. Furthermore, a role of T-helper 1 type cytokines such as INFγ, IL-2 and TNFβ has been suggested [4]. In addition, the immune modulator imiquimod that is used for topical treatment of BCC results in a massive peri- and intratumoral increase in macrophages [5].

In the skin, several types of antigen-presenting immune cells exist. In the mouse these are mainly macrophages and DC. DC that populate the basal and suprabasal layers of the epidermis are often called Langerhans cells (LC), while DC in the dermis are often called dermal DC (dDC) [6], [7]. Common to these cells is the potential to internalize particulate macromolecules and to display migratory properties [8]. In addition, these cells can express MHCII and/or F4/80 [6].

There is growing evidence that interactions between tumor cells and the surrounding stroma plays an important role in tumor progression. Several reports have demonstrated that DC including LC are essential in the generation of an antitumor immunity in the skin (e.g. [9], [10]). Given the rich network of these cells in the skin they are often thought to be the first immune cells to encounter tumor antigens from cutaneous cancers (for review see [11]). This also has been demonstrated for macrophages in extra-cutaneous cancers where macrophages can exert a beneficial role due to cytotoxicity to tumor cells but not to normal cells [12]. However, high macrophage numbers also have been linked to reduced survival of patients with solid cancers, indicating that the presence of macrophages could also be advantageous for tumor growth and metastasis [13]. Therefore, macrophages probably can have contrasting roles in cancer depending on their phenotype (for review see [14]).

Using the conditional *Ptch^flox/flox^ERT2^+/−^* knockout mouse model for BCC [15], we now provide evidence that macrophages and DC are involved in growth restraining processes in this tumor entity.

## Materials and Methods

### Mice and induction of BCC

Animals were sacrificed at the indicated end points of the experiments or if necessary due to the health conditions (e.g. weight loss >15%, apathy) by CO2 anesthesia followed by cervical dislocation. The mice used in the study were handled in accordance with the German animal protection law and the experiments were approved by the Niedersächsisches Landesamt für Verbraucherschutz und Lebensmittelsicherheit (permit numbers: 33.14.42502-04-026/09 and 33.14.42502-04-111/09). All experiments using animals were performed in compliance with all legal and ethical requirements. *Ptch^flox/flox^* mice have *loxP* sites in *Ptch* introns 7 and 9 [16]. *Rosa26CreERT2(ERT2)* mice express a tamoxifen-inducible cre recombinase under the control of the endogenous and ubiquitously expressed *Rosa26*-promoter [17]. *Ptch^flox/flox^ERT2^+/−^* mice were on a mixed C57BL/6 x Balb/c background. Genotyping of the *Ptch^flox^* and the Cre-mediated *Ptch^del^* alleles and of *ERT2* was performed as described [18]. All primers used for genotyping are listed in Table S1.

BCCs were induced in 8 week-old conditional *Ptch^flox/flox^ERT2^+/−^* mice by intramuscular (i.m.) injection of 100 μg tamoxifen as described [18], [19].

### Depletion of macrophages in mice using clodrolip

Liposome-encapsulated clodronate termed clodrolip was essentially prepared as described previously [20]. For depletion of macrophages in mice, clodrolip was injected intraperitoneally (i.p.) in *Ptch^floxflox^ERT2^+/−^* mice (n = 7) 15 days after tamoxifen-mediated BCC induction. The initial clodronate dose was 2 mg/20g body weight. Subsequently clodrolip was injected every fourth day at a dose of 1 mg/20 g and the treatment was continued for 75 days. The same amount of empty liposomes served as a control (n = 4). Clodrolip and empty liposomes were freshly diluted each time in PBS to obtain the desired drug dose in 120 μl for each animal. Animals were sacrificed 24 h after the last clodrolip or liposome dose. Spleen and skin samples were excised. Parts of the samples were either used for FACS analyses or RNA isolation, or were formalin-fixed and embedded in paraffin for immunohistological analyses.

### Cell culture experiments

The murine BCC cell line ASZ001 was established from UV-induced BCC of *Ptch* mutant mice and was cultured as described [21].

Cell viability/metabolic activity of ASZ001 was determined by addition of WST-1 reagent (Roche Diagnostics, Mannheim, Germany) according to the manufacturer's recommendations after incubation with 0.1 mg/ml clodrolip or the same amount of empty liposomes for 24–72 h.

### FACS analyses of tissue macrophages

FACS analysis of immune cells was performed on single cell suspensions of skin that were obtained as recently described [22]. Cells (1×10^6^) were stained with monoclonal antibodies against Mac1 (anti CD11b-FITC, BD Biosciences #557396) and F4/80 (anti F4/80-Cy5, eBiosciences 15-4801). At least 2×10^5^ viable cells were acquired on the basis of forward and side scattering and quantified by using a BD LSRII cytometer. Data acquisition and analysis were performed using the software BD FacsDiva (BD Biosciences Pharmingen) and FlowJo (Treestar, Ashland, OR).

### Gene expression analysis

Total RNA from skin was isolated using the RNeasy Fibrous Tissue Mini Kit (Qiagen, Hilden, Germany) and cDNA was synthesized using Superscript II and random hexamers (Invitrogen, Karlsruhe, Germany). Gene expression was analyzed by SYBR-green-based qRT-PCR assays on the ABI Prism HT 7900 Detection System instrument and software (Applied Biosystems, Darmstadt, Germany). The data was analyzed using the standard curve method for relative quantification. All primer pairs were intron-flanking and are shown in the Table S1. Amplification of *18S rRNA* served to normalize the amount of sample cDNA. Each sample was reverse transcribed twice and analyzed in triplicates. The mean value of each sample was used for analysis.

### Immunohistochemistry

Formalin-fixed tail skin was embedded in paraffin and sectioned at 5 μm for histological analyses. The identity of BCC was confirmed by examination of hematoxylin and eosin (H&E) stained sections. The paraffin sections were stained using a monoclonal anti-MHCII antibody (rat anti-mouse I-A/I-E #107602 from BioLegend; for antigen retrieval boric acid was used) that detects DC and to a lesser extend macrophages. In addition, an anti-F4/80 antibody (rat anti mouse #MCA497GA from Serotec; no antigen retrieval necessary) was employed that labels macrophages and to a lesser extend DC [6]. A biotinylated polyclonal antibody from Dako (# E0468, rabbit anti rat) served as secondary antibody. Following incubation with Streptavidin/HRP (#P0397, from Dako) and several washing steps, 3-Amino-9-Ethylcarbazol was used as a substrate. For Ki67 stainings the anti-Ki76 antibody #556003 from BD Pharmingen was used (antigen retrieval with citrate buffer pH 6). For detection of the primary antibody the Envision Polyclonal rabbit/mouse HRP kit (Dako, #K5007) was used. For TUNEL staining the DeadEnd Colorimetric TUNEL System (Promega, Germany) was applied according to the manufacturer's instructions. Staining was visualized with 3-Amino-9-Ethylcarbazol. After counterstaining with haematoxylin the sections were covered using glycergel (Dako GmbH, Hamburg).

MHCII^+^ cells were counted separately both in the epidermis (representing LC) and the dermis (representing mostly dDC) of 7 clodrolip treated and 4 liposome treated mice. F4/80^+^ cells were counted in the BCC stroma of 4 clodrolip treated and 3 liposome treated mice. For this purpose 2 independent paraffin-embedded skin samples (sample length and width app. 2.0 cm and 0.1 cm, respectively) from each mouse were sectioned and positive cells were counted on at least 3 microscopic fields of vision per sample. The analyzed stromal or epidermal areas (at least 3.6 mm^2^/field of vision or 1 mm^2^/field of vision, respectively) were measured using the software cellSens Dimension (*Olympus* Soft Imaging Solutions GmbH; Germany). Given are the numbers of F4/80 positive cells per mm^2^ stroma and MHCII positive cells per mm^2^ stroma or epidermis.

All Ki67^+^ cells of at least 1000 BCC cells on 2 independent paraffin sections of 7 clodrolip treated and 4 liposome treated mice were counted and the percentage of Ki67^+^ BCC cells was calculated.

TUNEL^+^ BCC cells were very rare. Thus all TUNEL^+^ cells of 2 independent paraffin sections each of 7 clodrolip treated and 4 liposome treated mice were counted.

Goldner staining was performed to visualize components of the connective tissue in the tumor stroma as described by Goldner [23]. This staining labels collagen in green, cell nuclei in brownish-red, and keratin in red color. Since most cells embedded in collagen are fibroblasts, calculation of the number of cell nuclei embedded in the collagen allows for quantification of fibroblasts localized in the tumor-adjacent stroma. In order to compare the amount of fibroblasts in liposome- and clodrolip-treated BCC-bearing skin (n = 4 and n = 7, respectively), 16 and 23 fields of vision (0.4 mm^2^ each) were analyzed, respectively. The stromal area (in green) was measured using the software cellSens Dimension (*Olympus* Soft Imaging Solutions GmbH; Germany). Subsequently, all cellular nuclei in this region were counted and MHCII^+^ and F4/80^+^ cells (counted on adjacent slides) were subtracted. Given is the number of cells per mm^2^ stroma.

### Measurement of tumor size

BCC size was measured on H&E stained sections using the area calculation tool of the software cellSens Dimension (*Olympus* Soft Imaging Solutions GmbH; Germany). For this purpose 6 independent skin samples (each sample at app. 2.0 cm length and 0.1 cm width) of 7 clodrolip treated and 4 liposome treated mice were sectioned at 5 μm. The tumor area was calculated by summing up the individual tumor area measured from each sample and consecutive normalization to the total skin area analyzed.

### Statistical analyses

If not otherwise indicated, statistical differences were analyzed using Mann-Whitney testing. Data was considered significant when *P*<0.05.

## Results

### Stromal areas in BCC-bearing skin of *Ptch^flox/flox^ERT2^+/−^* mice are infiltrated with MHCII^+^ cells

**Figure 1:**
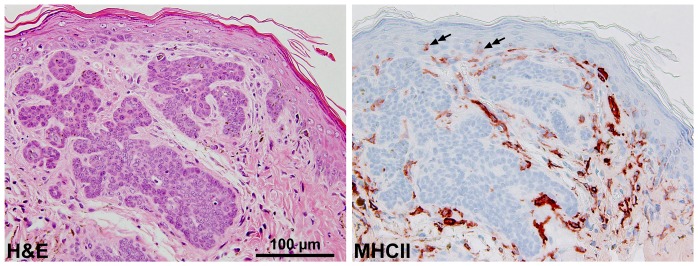
MHCII expression in BCC-bearing skin of *Ptch^flox/flox^ERT2^+/−^* mice. H&E staining and immunohistochemical analysis of MHCII expression on paraffin sections derived from BCC-bearing skin of *Ptch^flox/flox^ERT2^+/−^* mice. The secondary antibody alone did not result in any staining, hence the MHCII signals were specific (data not shown). double arrows: intraepidermal MHCII^+^ LC.

We recently showed that the stroma of BCC of *Ptch^flox/flox^ERT2^+/−^* mice is infiltrated with F4/80^+^ tumor-associated macrophages [19]. Using paraffin sections, we first confirmed this observation and stained paraffin embedded tissue sections with a MHCII antibody that marks dDCs and also LC in the murine skin and to lesser extent cutaneous macrophages [6]. As shown in Figure 1, the BCC stroma shows many MHCII^+^ cells, indicating that the tumor-bearing skin is abundantly infiltrated with the above-mentioned cell populations.

### Systemic clodrolip treatment of *Ptch^flox/flox^ERT2^+/−^* mice results in depletion of macrophages, dDC and LC and in an enhancement of BCC growth

In order to determine whether the network of cutaneous DC and macrophages are involved in growth-regulating processes in BCC we depleted these cells in BCC-bearing *Ptch^flox/flox^ERT2^+/−^* mice by treating the animals with clodrolip. Empty liposomes were used as vehicle-control. The treatment scheme was as previously published by Zeisberger and colleagues [20] and was started 15 days after tamoxifen-mediated induction of BCC. The treatment was continued for 75 days.

Successful macrophage depletion was first demonstrated by reduced F4/80 immunoreactivity of the spleens of clodrolip treated mice (Figure S1). Next we performed FACS analysis of single cell suspensions of the skin using Mac1 and F4/80 antibodies. Whereas Mac1 is expressed on granulocytes, T-, B- and NK-cells, DC and monocytes [24], macrophages are double positive for Mac1 and F4/80 [25]. For the analyses 3 clodrolip- and 3 vehicle-treated animals were sacrifized. As shown in Table 1, the numbers of Mac1^+^, F4/80^+^ and Mac1^+^F4/80^+^ double positive cells were generally lower in the skin of the clodrolip-treated cohort when compared to the controls (Table 1). As also shown in Table 1, the depletion was highly efficient in two of the clodrolip-treated mice (mouse 2 and mouse 3 in Table 1). These results were similar to those reported by Zeisberger and colleagues who showed that clodrolip treatment caused depletion of F4/80^+^ and Mac1^+^ cells [20].

**Table 1:** Systemic application of clodrolip decreases the numbers of macrophages in the skin.

		BCC-bearing skin		
	mouse	Mac1^+^	F4/80^+^	Mac1^+^F4/80^+^
**liposomes**	M1	5.98	4.30	4.40
	M2	4.97	4.82	3.57
	M3	3.02	3.77	2.52
	mean (SEM)	4.66 (0.87)	4.30 (0.30)	3.50 (0.54)
**clodrolip**	M1	4.69	2.41	2.38
	M2	1.00	0.82	0.64
	M3	1.74	0.96	0.85
	mean (SEM)	2.48 (1.13)	1.40 (0.51)	1.29 (0.55)

The percentage of Mac1^+^, F4/80^+^ and Mac1^+^F4/80^+^ double-positive cells in tumor-bearing skin of *Ptch^flox/flox^ERT2^+/−^* mice was determined by FACS. M1 to M3: mouse 1 to mouse 3.

**Figure 2:**
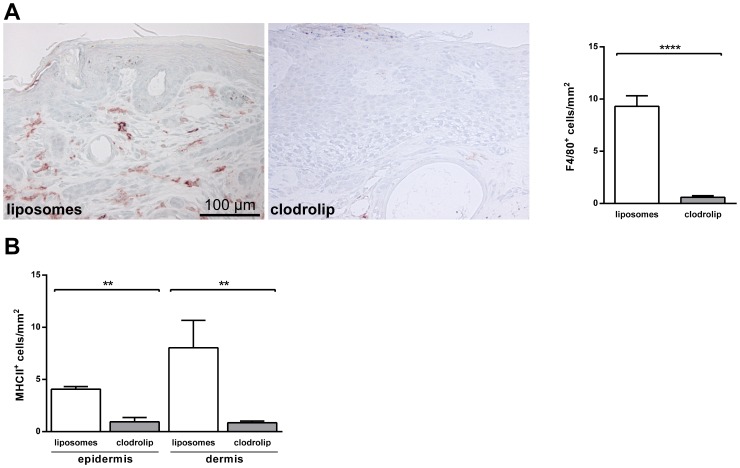
Clodrolip treatment results in depletion of cutaneous phagocytic cells in BCC-bearing skin of *Ptch^flox/flox^ERT2^+/−^* mice. (A) Immunohistochemical analysis of F4/80 expression on paraffin-sections of BCC-bearing skin derived from liposome- or clodrolip-treated *Ptch^flox/flox^ERT2^+/−^* mice. Left panel shows micrographs; right panel shows the absolute number of F4/80^+^ cells per square millimeter of BCC-bearing tissue. **** *P*<0.0001 (mean +SEM). (B) Absolute numbers of MHCII^+^ cells per square millimeter of BCC-bearing tissue as counted on stained paraffin-sections from liposome- or clodrolip-treated *Ptch^flox/flox^ERT2^+/−^* mice. MHCII^+^ cells were counted separately both in the epidermis (representing LC) and the stroma (representing DC). ** *P*<0.01.

A reduction of F4/80^+^ cells in the clodrolip-treated cohort was also revealed by immunohistochemistry (Fig. 2A). In addition, MHCII^+^ cells in the BCC stroma were almost entirely depleted in the clodrolip-treated group when compared to the vehicle-treated group (Fig. 2B). The clodrolip-treatment also resulted in a significant decrease in epidermal MHCII^+^ LC (Fig. 2B). Together these results show that clodrolip-treatment resulted in an efficient depletion of macrophages, dDC (i.e. DC in the dermis) and LC (i.e DC in the basal and suprabasal layers of the epidermis). With respect to macrophages and dDC the results are in accordance with the current literature, which describes depletion of these cells by clodronate. However, they are in contrast to published data showing that LC are resistant towards the drug [26]–[28].

**Figure 3:**
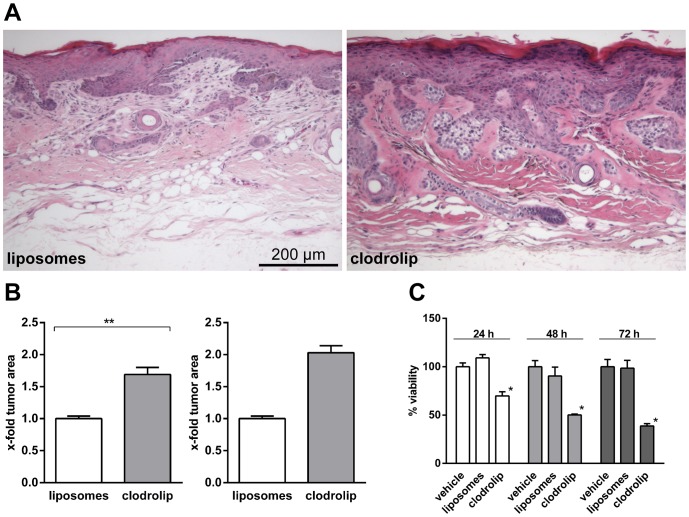
Clodrolip enhances BCC growth in *Ptch^flox/flox^ERT2^+/−^* mice. (A) H&E stainings of paraffin-sections derived from BCC-bearing skin of liposome- or clodrolip-treated *Ptch^flox/flox^ERT2^+/−^* mice. (B) Relative tumor areas of H&E-stained BCC-bearing skin samples of liposome- or clodrolip-treated *Ptch^flox/flox^ERT2^+/−^* mice. The mean value of the liposome-treated controls was normalized to 1. Left panel shows the mean value (+SEM) of all liposome- and clodrolip-treated animals **P<0.01; right panel shows the mean value (+SEM) of all liposome-treated animals in comparison with 2 clodrolip-treated mice (mouse 2 and mouse 3 in Table 1), in which the phagocytic cells have been very efficiently depleted (due to the small sample size, statistical analysis was omitted). (C) Viability/metabolic activity of the BCC cell line ASZ001 after treatment with PBS, liposomes or clodrolip. Mean value of the PBS-treated controls was set to 100%. **P<0.01.

Next, we studied the effects of clodrolip on BCC growth. For this purpose the BCC area was analyzed on H&E stained paraffin sections derived from 7 clodrolip- and 4 vehicle-treated mice. The tumor area was calculated by summing up the individual tumor area measured from each sample (see material and methods) and normalization to the total area of the skin analyzed. The data revealed that the BCC of the clodrolip-treated group were significantly enlarged when compared to the vehicle-treated group (Fig. 3A; Fig. 3B left panel). This was also obvious when comparing the BCC size of the vehicle-treated group with that of the 2 animals (mouse 2 and mouse 3 in Table 1) in which the clodrolip-treatment resulted in efficient depletion of macrophages in the skin (Fig. 3B right panel).

In order to examine whether clodrolip or empty liposomes directly influenced tumor cell growth, we also investigated the toxic effects of clodrolip on BCC cells *in vitro*. For this purpose, the BCC cell line ASZ001 was incubated with 0.1 mg/ml clodrolip for 24 h to 72 h. The same amounts of empty liposomes or of PBS alone were used as controls. As shown in Figure 3C clodrolip significantly reduced the viability of ASZ001 when compared to the controls (Fig. 3C). These data indicate that clodrolip has direct tumoricidal activity. Therefore the *tumor-promoting* activity of clodrolip *in vivo* might be an indirect effect of the drug on the tumor cells.

Together these data show that macrophages and DC including LC in BCC-bearing skin were efficiently depleted after treatment with clodrolip. This depletion was accompanied by a significant enhancement of tumor growth.

### Clodrolip treatment of BCC-bearing skin of *Ptch^flox/flox^ERT2^+/−^* mice decreases the number of collagen-producing stromal cells

**Figure 4:**
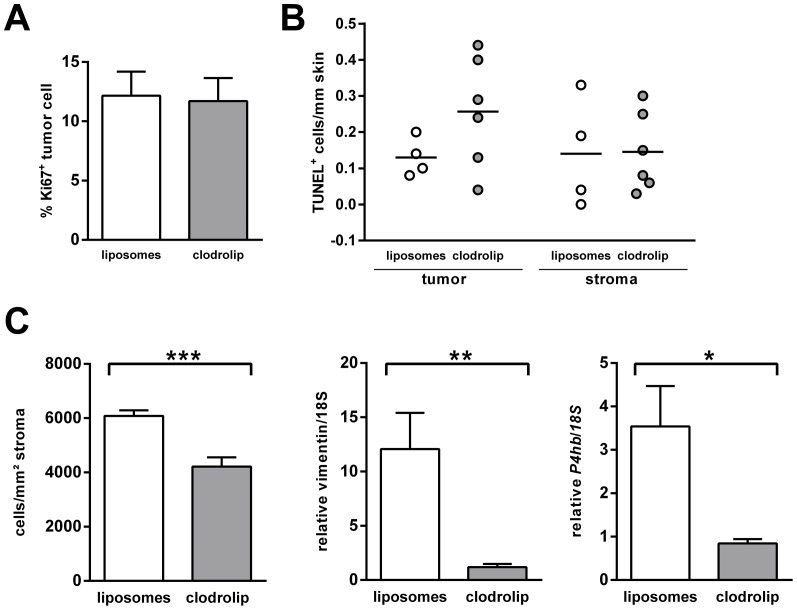
Clodrolip decreases the number of collagen-producing cells and the mRNA expression of the fibroblast markers *vimentin* and *P4hb* in BCC-bearing skin of *Ptch^flox/flox^ERT2^+/−^* mice. (A) Percentage of Ki67^+^ BCC cells in liposome- or clodrolip-treated *Ptch^flox/flox^ERT2^+/−^* mice. (B) TUNEL^+^ BCC cells per mm BCC-bearing skin in liposome- or clodrolip-treated *Ptch^flox/flox^ERT2^+/−^* mice. (C) Collagen-producing cells (mainly fibroblast) as estimated by Goldner staining (left panel; see main manuscript for detailed informations) and qRT-PCR analysis of *vimentin* (middle panel) and of *P4hb* (right panel) in BCC-bearing skin of *Ptch^flox/flox^ERT2^+/−^* mice treated with liposomes or clodrolip. Data shows the mean value (+SEM). * *P*<0.05, ** *P*<0.01, *** *P*<0.001.

In order to analyze whether the clodrolip-mediated enhancement of BCC growth was associated with a decrease in proliferation or with increased apoptosis, we performed immunohistochemical analysis of the proliferation marker Ki67 and TUNEL staining, respectively. However, neither the numbers of Ki67^+^ nor of TUNEL^+^ cells were different between the cohorts (Figure 4A and 4B, respectively). In addition, a first expression analysis of the differentiation marker *K10* that is solely expressed by epidermal cells did not reveal any significant difference (data not shown). However, we found that the clodrolip treatment resulted in a decrease in collagen-producing stromal cells. This became obvious by a Goldner staining (see Figure S2). As shown in Fig 4C (left panel) the number of collagen-producing cells that are mainly fibroblasts was significantly lower in the clodrolip-treated cohort when compared to the liposome-treated cohort. This was in line with qRT-PCR data showing that the expression of the fibroblast markers *vimentin* and *prolyl-4-hydroxylase beta* (*P4hb*) was decreased in the clodrolip-treated samples (Fig. 4C, middle and right panel). Together, these data suggest that the clodrolip-treatment, besides lowering the F4/80^+^ and MHCII^+^ cells in the tumor microenvironment, also decreases the numbers of collagen-producing cells, which are in all likelihood stromal fibroblasts.

## Discussion

Our data suggest that cutaneous macrophages and DC are necessary for tumor growth restraining processes in the *Ptch^flox/flox^ERT2^+/−^* mouse model for BCC. This assumption is based on the observations that the BCC stroma is infiltrated with these cells and that a clodronate-liposome mediated depletion enhances BCC growth. In addition, the clodrolip treatment was accompanied by a decrease in collagen-producing cells. Because the fibroblast markers *vimentin* and *P4hb* were downregulated as well, it is likely that the respective cells were fibroblasts. The reduction of fibroblasts was probably not a direct toxic effect of clodronate because i) liposome-encapsuled clodronate is not internalized by non-phagocytic cells [29] and ii) clodronate in cell culture only marginally affects fibroblast growth [30]. Instead, the decrease of the collagen-expressing cells might be directly associated with the loss of macrophages that have been shown to enhance fibroblast proliferation [31], probably by the secretion of TNFα, PDGF or IL-1β [31], [32]. However, these assumptions are pure speculations and need more investigation in the future.

Together, our experiments demonstrate a very important role of macrophages and DC in growth restraining processes in BCC. In our experiments application of liposome-encapsulated clodronate resulted in effective depletion of these cells as demonstrated by loss of MHCII^+^ cells that is expressed by professional immune antigen-presenting cells. In addition depletion of macrophages was also demonstrated by loss of Mac1^+^F4/80^+^ cells. This is in accordance to other reports that demonstrated elimination of DC and macrophages upon treatment with clodronate [26], [33]. We observed a significant reduction of epidermal LC. This is in contrast to other reports showing that LC are resistant to clodronate [28]. However, since the skin in our setting was highly loaded with BCC it is possible that the LC were activated and thus phagocytosed this toxic drug at higher avidity.

The clodronate-liposome mediated loss of DC and the accompanying enhancement of BCC growth in our model match the established role of these cells in non-melanoma cutaneous cancer. Thus, specific subsets of DC inclusive LC are beneficial for tumor eradication in the skin. This is due to their ability to either recognize, process and present foreign (tumor) antigens to T cells or to stimulate T cell proliferation and enhance T cell activity, respectively. In addition, DC including both LC and dDC can produce IFN-α in response to foreign (tumor) antigens. Accordingly, it has been shown that elevated amounts of dDC subsets are indeed associated with increased clearance of BCC lesions following treatment with imiquimod (for review see [11]). In addition to dDC and LC, macrophages can play beneficial roles in cancer [34]. Thus, in contrast to tumor-promoting M2 macrophages, these so-called M1 macrophages act by defending the host from infections and also from tumors. Therefore, depletion of the latter cells can result in tumor-promoting effects, which also may have contributed to the enhancement of BCC growth in our model as well.

In the clinics, bisphosphonates such as clodronate are frequently used to delay the spread of bone metastases. This is due to the fact that bisphosphonates strongly bind to mineralized bone surfaces and are ingested by osteoclasts, wherein they inhibit osteolysis, thereby preventing the release of growth factors from the bone matrix [35]. In addition, bisphosphonates also revealed direct killing of different cancer cells, including myeloma, breast cancer, prostate cancer, and lung cancer *in vitro* (reviewed in [36]). Indeed, also in our experiments the drug efficiently decreased the viability of the BCC cell line ASZ001. Furthermore, bisphosphonates encapsulated in nanocarriers such as liposomes can inhibit angiogenesis and can deplete tumor-promoting macrophages, whereas the free drug mainly inhibits osteolysis. In line with all these effects many studies on a variety of tumors thus report that administration of bisphosphonates generates an antitumor activity [36]. However, despite these data there are also several reports showing *protumoral* effects of clodronate administered as free drug. For example, a study of 299 women with breast cancer demonstrated a significantly increased rate of non-skeletal metastases and a significantly lower overall survival rate after clodronate therapy [37]. Furthermore, cases of esophagus carcinoma have been described after oral bisphosphonate use [38]. These studies show that, as in our study, clodronate can also result in enhancement of cancerous malignancies.

In summary, in our BCC model the tumor-promoting effects of liposome-encapsulated clodronate may result from various processes. These include a decrease in antigen-presenting cells of the skin (i.e. of DC including LC and macrophages), a decrease in collagen-producing cells and most likely other so far unknown molecular processes such as modulation of T cell numbers, modulation of dermal angiogenesis, and the induction of a wide palette of cytokines such as IFNγ, IL-17, IL-1, IL-6, TNFα and IL-23 that are all modulated in the skin upon treatment with clodronate [39]. An elucidation of the exact mechanisms underlying the tumor-promoting effects of clodrolip presented here will be further complicated by the fact that empty liposomes themselves can induce antitumor effects (see Figure S3) that are likewise associated with macrophage responses such as e.g. the induction of *iNOS*, *Arg1* and *Trem2* [22]. Nevertheless, our data strongly suggest that macrophages and DC of the skin play an important role in skin cancer pathogenesis in that they exert antitumor effects on BCC, the most common tumor in humans.

## Supporting Information

Figure S1 **Depletion of F4/80-expressing cells in spleens derived from clodrolip-treated** ***Ptch^flox/flox^ERT2^+/−^*** **mice.** Immunohistochemical analysis using an anti-F4/80 antibody of paraffin-embedded spleens of *Ptch^flox/flox^ERT2^+/−^* mice treated with empty liposomes or clodrolip.
(DOCX)

Figure S2 **Evaluation of amounts of fibroblasts by the Goldner method.** The Goldner method labels collagen in green and the nuclei and keratin in red color. Calculation of the number of cell nuclei embedded in the collagen (in the lower panel, the coloring of the tumor-bearing epidermis was attenuated using the software FreeHand MX (Macromedia Inc.) to better visualize the collagen-producing stromal cells) allows for quantification of fibroblasts localized in the tumor-adjacent stroma. As demonstrated by the figures, the amount of nuclei embedded in the collagen is lower in the clodrolip-treated BCC when compared to the liposome-treated BCC.
(DOCX)

Figure S3 **Empty liposmes themselves can induce antitumor effects in BCC-bearing** ***Ptch^flox/flox^ERT2^+/−^*** **mice.** HE-stained skin sections of liposome-treated mice (left panel) and PBS-treated mice (right panel). The analysis of the tumor-bearing skin revealed that tumors of PBS-treated mice are larger when compared to liposome-treated mice.
(DOCX)

Table S1 **Oligonucleotides used for genotyping and reverse-transcription polymerase chain reaction.**
(DOCX)
