# Supplementary material for: Depletion of Cutaneous Macrophages and Dendritic Cells Promotes Growth of Basal Cell Carcinoma in Mice
Source: PLoS One. 2014 Apr 1;9(4):e93555. doi: 10.1371/journal.pone.0093555 (PMC3972151; doi:10.1371/journal.pone.0093555)
Supplement: Figure S1 — Depletion of F4/80-expressing cells in spleens derived from clodrolip-treated Ptchflox/floxERT2+/− mice. Immunohistochemical analysis using an anti-F4/80 antibody of paraffin-embedded spleens of Ptchflox/floxERT2+/− mice treated with empty liposomes or clodrolip. (DOCX) [file pone.0093555.s001.docx]

**Supplementary Figure S1:**

**Depletion of F4/80-expressing cells in spleens derived from clodrolip-treated *Ptch^flox/flox^ERT2^+/−^* mice.** Immunohistochemical analysis using an anti-F4/80 antibody of paraffin-embedded spleens of *Ptch^flox/flox^ERT2^+/−^* mice treated with empty liposomes or clodrolip.

**
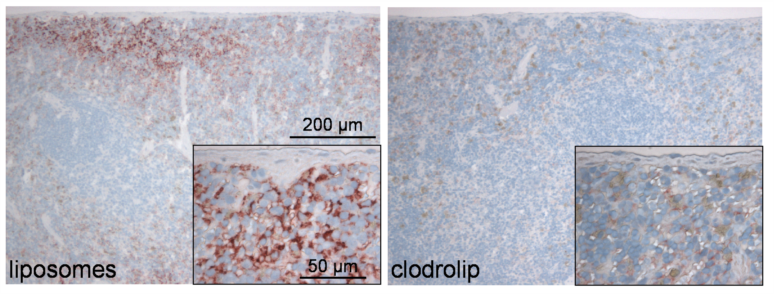
**
