# Supplementary material for: Depletion of Cutaneous Macrophages and Dendritic Cells Promotes Growth of Basal Cell Carcinoma in Mice
Source: PLoS One. 2014 Apr 1;9(4):e93555. doi: 10.1371/journal.pone.0093555 (PMC3972151; doi:10.1371/journal.pone.0093555)
Supplement: Figure S2 — Evaluation of amounts of fibroblasts by the Goldner method. The Goldner method labels collagen in green and the nuclei and keratin in red color. Calculation of the number of cell nuclei embedded in the collagen (in the lower panel, the coloring of the tumor-bearing epidermis was attenuated using the software FreeHand MX (Macromedia Inc.) to better visualize the collagen-producing stromal cells) allows for quantification of fibroblasts localized in the tumor-adjacent stroma. As demonstrated by the figures, the amount of nuclei embedded in the collagen is lower in the clodrolip-treated BCC when compared to the liposome-treated BCC. (DOCX) [file pone.0093555.s002.docx]

**Supplementary Figure S2:**

**Evaluation of amounts of fibroblasts by the Goldner method.** The Goldner method labels collagen in green and the nuclei and keratin in red color. Calculation of the number of cell nuclei embedded in the collagen (in the lower panel, the coloring of the tumor-bearing epidermis was attenuated using the software FreeHand MX (Macromedia Inc.) to better visualize the collagen-producing stromal cells) allows for quantification of fibroblasts localized in the tumor-adjacent stroma. As demonstrated by the figures, the amount of nuclei embedded in the collagen is lower in the clodrolip-treated BCC when compared to the liposome-treated BCC.

**
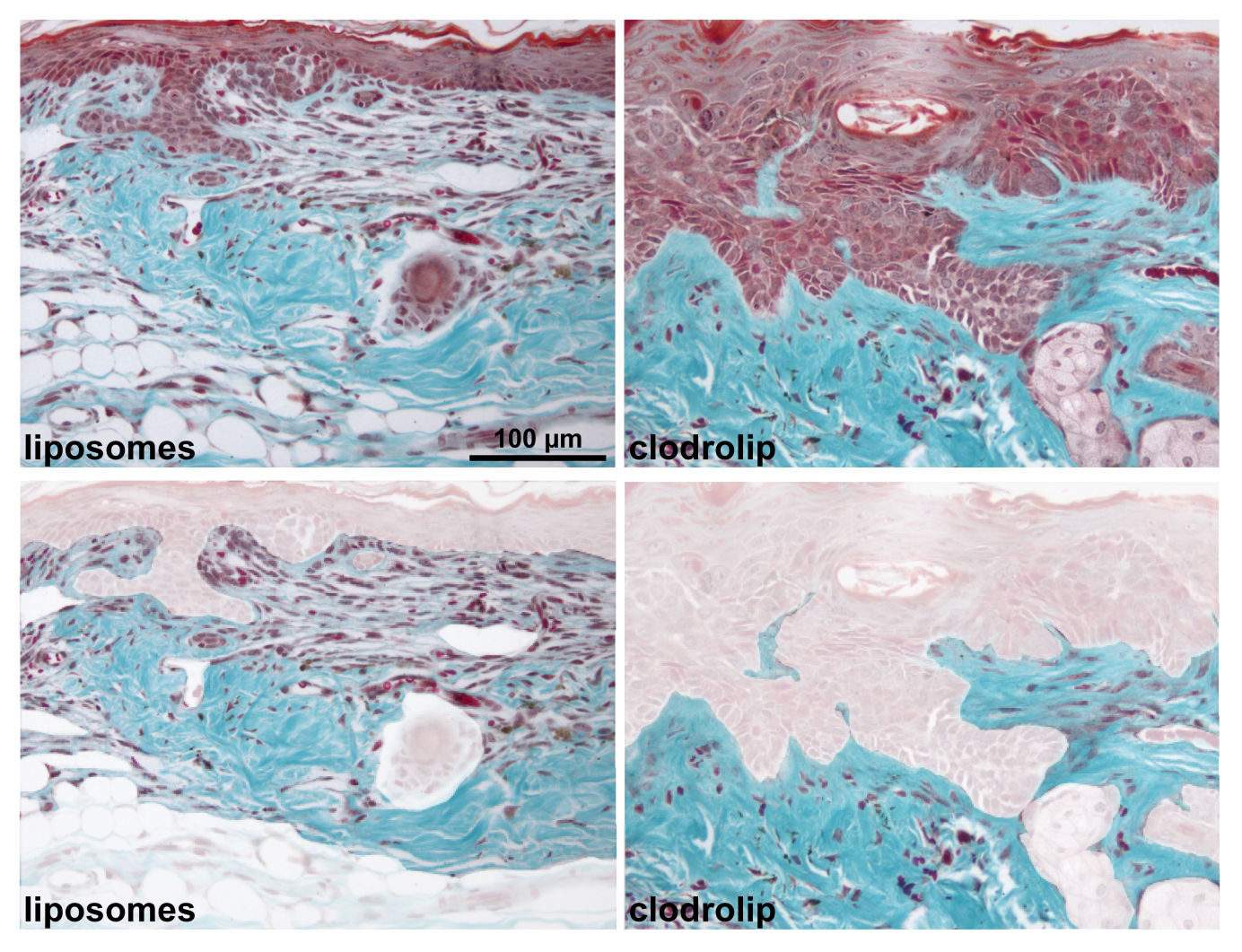
**
