# Supplementary material for: Depletion of Cutaneous Macrophages and Dendritic Cells Promotes Growth of Basal Cell Carcinoma in Mice
Source: PLoS One. 2014 Apr 1;9(4):e93555. doi: 10.1371/journal.pone.0093555 (PMC3972151; doi:10.1371/journal.pone.0093555)
Supplement: Figure S3 — Empty liposmes themselves can induce antitumor effects in BCC-bearing Ptchflox/floxERT2+/− mice. HE-stained skin sections of liposome-treated mice (left panel) and PBS-treated mice (right panel). The analysis of the tumor-bearing skin revealed that tumors of PBS-treated mice are larger when compared to liposome-treated mice. (DOCX) [file pone.0093555.s003.docx]

**Supplementary Figure S3:**

Empty liposmes themselves can induce antitumor effects in BCC-bearing *Ptch^flox/flox^ERT2^+/−^* mice. HE-stained skin sections of liposome-treated mice (left panel) and PBS-treated mice (right panel). The analysis of the tumor-bearing skin revealed that tumors of PBS-treated mice are larger when compared to liposome-treated mice

**
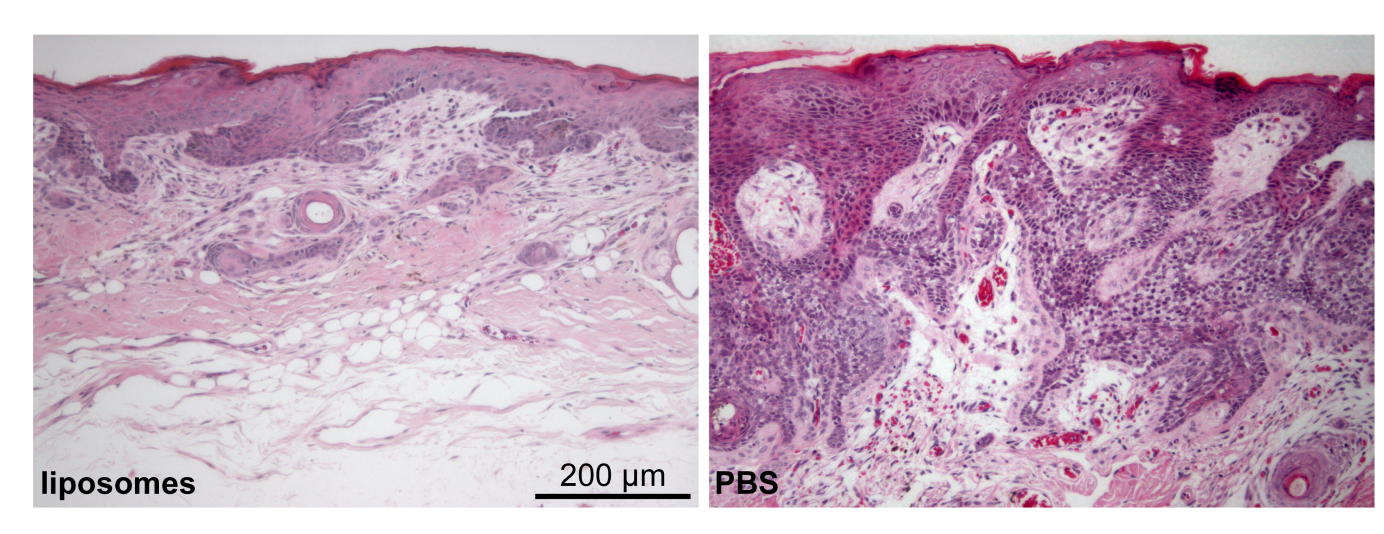
**
