# Supplementary material for: Depletion of Cutaneous Macrophages and Dendritic Cells Promotes Growth of Basal Cell Carcinoma in Mice
Source: PLoS One. 2014 Apr 1;9(4):e93555. doi: 10.1371/journal.pone.0093555 (PMC3972151; doi:10.1371/journal.pone.0093555)
Supplement: Table S1 — Oligonucleotides used for genotyping and reverse-transcription polymerase chain reaction. (DOCX) [file pone.0093555.s004.docx]

**Supplementary Table S1: Oligonucleotides used for genotyping and reverse-transcription polymerase chain reaction**

| **Genotyping** | | |
| --- | --- | --- |
| **Primer Name** | **Primer Sequence (5’-3’ orientation)** | **Application** |
| 9260 | GAGGAGAAGCGCAGTCAATC | *Wnt5a* knockout mice and BMDM |
| 9261 | CATCTCAACAAGGGCCTCAT |  |
| oIMR7415 | GCCAGAGGCCACTTGTGTAG |  |
| mPTCNx_f | TGGTAATTCTGGGCTCCCGT | *Ptch^flox/flox^* mice |
| mPTCNx_r | CCGGTAGAATTAGCTTGAAGTTCCT |  |
| mPTCwt_r.2 | ACACAACAGGGTGGAGACCACT |  |
| Exon7-F | AGGAAGTATATGCATTGGCAGGAG | recombination of the Ptch locus in *Ptch^flox/flox^ERT2^+/-^* mice |
| neo-R | GCATCAGAGCAGCCGATTGTCTG |  |
| Ella-Cre-F | CCAGGCTAAGTGCCTTCTCTACA | *ERT2^+/-^* knockout mice |
| Ella-Cre-R | AATGCTTCTGTCCGTTTGCCGGT |  |

| **Semiquantitative/Quantitative reverse-transcription polymerase chain reaction** | | |
| --- | --- | --- |
| **Primer Name** | **Primer Sequence (5’-3’ orientation)** | **Application** |
| 18S-fwd | CGCAAATTACCCACTCCCG | murine *18s* expression analysis (qRT-PCR) |
| 18S-rev2 | TTCCAATTACAGGGCCTCGAA |  |
| mVim-F2 | AAAGCACCCTGCAGTCATTC | murine *vimentin* expression analysis (qRT-PCR) |
| mVim-R2 | GCTCCTGGATCTCTTCATCG |  |
| mP4hb-F | GTTCTCCAAGTACCAGCTGGACA | murine *P4hb* expression analysis (qRT-PCR) |
| mP4hb-R | GACCAAAGGCAGCTGATTGTGCT |  |
| mK10-F | GGATGCTGAAGAGTGGTTCAA | murine *K10* expression analysis (qRT-PCR) |
| mK10-R | TCTGTTTCTGCCAAGGAGGCT |  |
